# Small Molecule Modulators of the Circadian Molecular Clock With Implications for Neuropsychiatric Diseases

**DOI:** 10.3389/fnmol.2018.00496

**Published:** 2019-01-21

**Authors:** Hyo Kyeong Cha, Sooyoung Chung, Hye Young Lim, Jong-Wha Jung, Gi Hoon Son

**Affiliations:** ^1^Department of Biomedical Sciences, College of Medicine, Korea University, Seoul, South Korea; ^2^Department of Brain and Cognitive Sciences, Scranton College, Ewha Womans University, Seoul, South Korea; ^3^College of Pharmacy, Research Institute of Pharmaceutical Sciences, Kyungpook National University, Daegu, South Korea

**Keywords:** circadian rhythm, circadian clock, cryptochrome, REV-ERB, ROR, small molecule, circadian rhythm-related disease

## Abstract

Circadian rhythms regulate many biological processes and play fundamental roles in behavior, physiology, and metabolism. Such periodicity is critical for homeostasis because disruption or misalignment of the intrinsic rhythms is associated with the onset and progression of various human diseases and often directly leads to pathological states. Since the first identification of mammalian circadian clock genes, numerous genetic and biochemical studies have revealed the molecular basis of these cell-autonomous and self-sustainable rhythms. Specifically, these rhythms are generated by two interlocking transcription/translation feedback loops of clock proteins. As our understanding of these underlying mechanisms and their functional outputs has expanded, strategies have emerged to pharmacologically control the circadian molecular clock. Small molecules that target the molecular clock may present novel therapeutic strategies to treat chronic circadian rhythm-related diseases. These pharmaceutical approaches may include the development of new drugs to treat circadian clock-related disorders or combinational use with existing therapeutic strategies to improve efficacy via intrinsic clock-dependent mechanisms. Importantly, circadian rhythm disruptions correlate with, and often precede, many symptoms of various neuropsychiatric disorders such as sleep disorders, affective disorders, addiction-related disorders, and neurodegeneration. In this mini-review, we focus on recent discoveries of small molecules that pharmacologically modulate the core components of the circadian clock and their potential as preventive and/or therapeutic strategies for circadian clock-related neuropsychiatric diseases.

## Introduction

Circadian rhythms are ubiquitous biological oscillations with an approximate 24-h period. These evolutionarily well-conserved rhythms arise from an intrinsic timekeeping system known as the “circadian clock”, which allows organisms to anticipate environmental cycling and coordinate biological processes. This clock is self-sustainable through an elaborate cooperation of genetic components and is hierarchically organized into a circadian timing system. In mammals, the apex of this system is the suprachiasmatic nucleus (SCN) of the hypothalamus, which is considered the central or master clock (Ralph et al., 1990; Reppert and Weaver, 2002). The SCN integrates environmental cues such as light into time information to entrain its phase and then conveys this information to other oscillators in extra-SCN brain regions and peripheral tissues. Indeed, in multi-cellular organisms, most cells harbor cell-autonomous oscillators. These so-called local or peripheral clocks contribute to overt circadian rhythms, including the rest-activity cycle, periodic daily variations in metabolism and body temperature, as well as rhythmic hormone secretion (Dibner et al., 2010; Son et al., 2011).

Robust circadian timing is required for health, and disruption of these intrinsic rhythms causes diverse pathologies. For instance, circadian disruption caused by shift-work, jet-lag, or mis-timed food intake is considered a risk factor for various chronic diseases, including sleep disorders, metabolic syndromes, cardiovascular diseases, affective disorders, neurodegeneration, and tumorigenesis (Takahashi et al., 2008; Bechtold et al., 2010). To develop treatments for these disorders, extensive studies have identified several small molecule compounds that can directly modulate circadian clocks. In this mini-review, we will discuss recent investigations of the most promising of these small chemical compounds and their therapeutic implications in neuropsychiatric diseases.

## The Mammalian Circadian Molecular Clock

**FIGURE 1:**
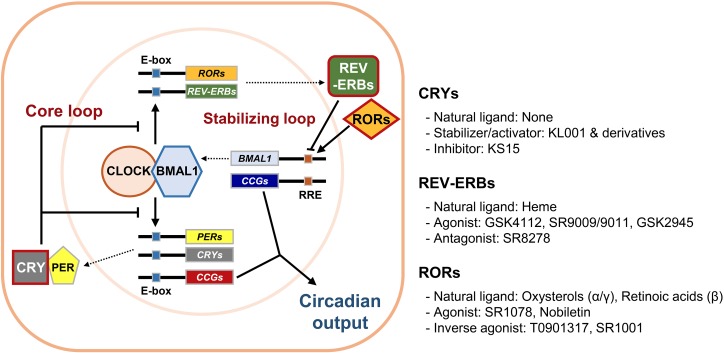
The mammalian circadian molecular clock and its potential drug targets. The mammalian circadian clock is composed of two interlocking transcription/translation feedback loops, the core and stabilizing/auxiliary loops, respectively. The integral components of the core loop are CLOCK (or NPAS2) and BMAL1, which form a heterodimer and then induce E-box-mediated transcription of their negative regulators *Period*s (PERs) and *Cryptochrome*s (CRYs). Accumulated PER and CRY proteins repress E-box-mediated transcription until they are sufficiently cleared by proteasome-mediated degradation. CLOCK and BMAL1 also control expression of circadian nuclear receptors such as RORs and REV-ERBs, which modulate *Bmal1* mRNA levels by competitive actions on the RORs/REV-ERBs-responsive elements (RREs) in the *Bmal1* promoter. Collectively, cycling of clock components determines the periodic mRNA expression levels of various clock-controlled genes (CCGs) through E-box, RRE, and/or other *cis*-elements recognized by secondary circadian transcription factors, thus generating rhythmic physiological outputs. Of these core clock proteins, we focused primarily on CRYs, REV-ERBs, and RORs (red boxes), which were recently identified as targets for small molecule modifiers of the circadian clock.

The self-sustainable nature of the circadian system is primarily attributed to circadian molecular oscillators. The molecular clock is composed of several clock proteins that are required for the generation and maintenance of cell-autonomous rhythms (Dibner et al., 2010). Clock proteins form two interlocking positive and negative transcription/translation feedback loops that drive periodic expression of their target genes (Figure 1). The primary regulators are Circadian Locomotor Output Cycle Kaput (CLOCK) and Brain Muscle Aryl Hydrocarbon Receptor Nuclear Translocator-Like 1 (BMAL1, encoded by the *ARNTL* gene). They belong to the basic helix-loop-helix–PER-ARNT-SIM (bHLH–PAS) transcription factor family. CLOCK and BMAL1 activate transcription of target genes by forming heterodimers and binding to E-box enhancer elements (5′-CACGTG-3′) in the promoter/enhancer regions. In addition to CLOCK, Neuronal PAS 2 (NPAS2) is another bHLH–PAS protein enriched in forebrain regions that can also form heterodimers with BMAL1 to control E-box element-dependent gene transcription (Asher and Schibler, 2006). The targets include proteins that form a negative feedback loop such as PERIODs (PERs: PER1, 2, and 3) and CRYPTOCHROMEs (CRYs: CRY1 and 2). Accumulated PER and CRY proteins form repressive complexes that suppress E-box-mediated transcription by binding to CLOCK/BMAL1 heterodimers, whereas PER and CRY degradation terminates this repression and reinitiates transcription (Gekakis et al., 1998; Hogenesch et al., 1998; Kume et al., 1999; Shearman et al., 2000). Stability of PER and CRY proteins is linked with their post-translational modifications and is crucial for proper circadian period length. It is well known that the *Tau*-mutant hamster, bearing a mutation in the casein kinase 1𝜀 (*CK1𝜀*) gene, displays a shortened free-running period in locomotor activities (Ralph and Menaker, 1988). In accordance, PER proteins are phosphorylated by CK1s prior to their proteasomal degradation, contributing to regulation of circadian period lengths (Eide et al., 2005; Shirogane et al., 2005). Similarly, CRY protein phosphorylation by adenosine monophosphate-activated protein kinase (AMPK) or glycogen synthase kinase 3β (GSK3β) leads to degradation mediated by paralogous F-box proteins, FBXL3 and FBXL21 (Busino et al., 2007; Lamia et al., 2009; Kurabayashi et al., 2010; Hirano et al., 2013; Yoo et al., 2013). Mutations in the *Fbxl3* gene result in long-period phenotypes in mice, whereas *Fbxl21*-mutant mice display short-period phenotypes (Godinho et al., 2007; Siepka et al., 2007; Hirano et al., 2013; Yoo et al., 2013). This CLOCK/BMAL1-initiated loop is considered the core loop of the mammalian clock.

An additional stabilizing loop adjusts the amounts of bHLH–PAS proteins. This secondary loop consists of sets of the circadian nuclear receptors, in particular REV-ERBs (REV-ERBα and β, encoded by *NR1D1* and *NR1D2*, respectively) and retinoic acid receptor-related orphan nuclear receptors (RORs: RORα-γ), that are also under the transcriptional control of CLOCK/BMAL1 heterodimers. REV-ERBs and RORs compete to occupy the RORs/REV-ERBs-responsive elements (RREs) located in the promoter/enhancer regions of their target genes. RORs usually activate RRE-mediated transcription, whereas REV-ERBs strongly suppress it (Preitner et al., 2002; Ueda et al., 2002; Sato et al., 2004). This stabilizing loop was originally considered as accessory because only moderate phenotypes were observed in mutant mice bearing null alleles of any of these genes. However, more recent studies using inducible double knockouts for both *Nr1d1* and *2* revealed that their compensatory activity yielded these subtle phenotypes and that REV-ERBs are required for normal period regulation (Cho et al., 2012). REV-ERBs also control circadian outputs by cooperating with cell type-specific transcriptional regulators (Chung et al., 2014; Zhang et al., 2015). Additional feedback loops involving the proline and acidic amino acid-rich basic leucine zipper proteins (PARbZip), such as D-box binding protein (DBP) and E4 promoter-binding protein 4 (E4BP4), as well as several members of bHLH transcription factors (BHLHE40 and BHLHE41), also intersect with the main loops to confer further regulation and mediate circadian expression of subsets of clock-controlled genes (Mitsui et al., 2001; Honma et al., 2002).

## Small Molecules Targeting Clock Proteins

As noted earlier, circadian disruptions are pivotal in various biological dysfunctions. Subsequent studies have attempted to correct these dysfunctions by exploring pharmacological strategies (Schroeder and Colwell, 2013). Initially, high-throughput screening studies identified several compounds that influence circadian oscillators by acting on post-translational regulators, including CK1s, CK2, GSK3β, and AMPK (Chen et al., 2018). These studies have advanced our understanding of the post-translational mechanisms underlying the circadian clock and uncovered novel clock-regulatory pathways. Additionally, some of the clock modulators that target these signaling pathways have already been recognized for their therapeutic implications (He and Chen, 2016; Chen et al., 2018). For example, lithium, a widely used mood stabilizer, inhibits GSK3β and lengthens the circadian period; however, some synthetic inhibitors exhibited opposite effects (Hirota et al., 2008; Li et al., 2012). Also, AMPK activators with a wide range of beneficial metabolic and physiological effects also altered circadian gene expression, as demonstrated both *in vivo* and *in vitro* (Um et al., 2007; Lamia et al., 2009). These observations suggest that modulation of the circadian clock may have beneficial effects on circadian rhythm-related chronic diseases. In this regard, recent investigations have attempted to directly target core components of the mammalian circadian clock by using small-molecule modifiers. Representative small molecules that bind to core clock components are summarized in Table 1. Pharmacological targets of these small molecules include CRYs, REV-ERBs, and RORs, which are described below.

**Table 1:** Representative small molecule clock modulators.

Name	Structure	Actions	Potential applications	Reference
KL001 and analogs	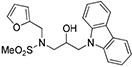	CRY stabilizer Suppresses E-box-mediated transcription Alters period	Metabolic disorders	Hirota et al., 2012; Nangle et al., 2013
KS15	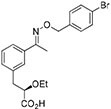	CRY inhibitor Enhances E-box-mediated transcription	Cancer	Chun et al., 2014, 2015; Jang et al., 2018
SR9009 and related compounds	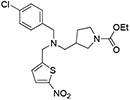	REV-ERB agonist Suppresses RRE-mediated transcription	**Sleep disorders Anxiety disorders** Metabolic disorders Cancer	Solt et al., 2012; Banerjee et al., 2014; Sulli et al., 2018b
SR8278	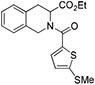	REV-ERB antagonist Enhances RRE-mediated transcription	**Depressive disorders Risk of bipolarity**	Kojetin et al., 2011; Chung et al., 2014; Guo et al., 2018
Nobiletin	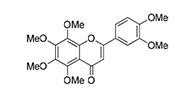	RORα/γ agonist Enhances RRE-mediated transcription Increases amplitude	**Depressive disorders Neurodegeneration** Metabolic disorders	Onozuka et al., 2008; Yi et al., 2011; Yabuki et al., 2014; Nakajima et al., 2015; He et al., 2016
SR1078 and related compounds	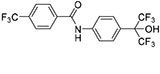	RORα/γ agonist Enhances RRE-mediated transcription	**Autism-spectrum disorders** Diabetic cardiomyopathy	Wang et al., 2010, 2016; Zhao et al., 2017
SR1001 and related compounds	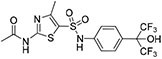	RORα/γ inverse agonist Suppresses RRE-mediated transcription	Metabolic disorders Atherosclerosis Autoimmunity Anti-inflammation	Solt et al., 2011; Billon et al., 2016; Dai et al., 2017


*CRY, cryptochrome; ROR, retinoic acid receptor-related orphan nuclear receptor; RRE, RORs/REV-ERBs-responsive element.*

### CRYs: Key Targets of Small Molecules That Act Directly on the Core Loop

A carbazole derivative, KL001, and its analogs are the first-in-class small molecules that target the core components of the mammalian clock (Hirota et al., 2012). In cultured SCN explants and fibroblasts, continuous treatment with these compounds significantly lengthens the circadian period and reduces amplitude of both *Bmal1* and *Per2* promoter activity, implying CRY protein activation. KL001 binds to CRY through the FAD-binding pocket, which is known to be recognized by FBXL3 and mediate proteasomal degradation (Xing et al., 2013). The co-crystal structure of the KL001–CRY2 complex revealed that KL001 competes with FAD and interferes with binding of the FBXL3 C-terminal to CRY, thereby stabilizing CRY proteins (Hirota et al., 2012; Nangle et al., 2013). Alternatively, we identified a derivative of 2-ethoxypropanoic acid (designated as KS15) that inhibits CRY-mediated feedback on CLOCK/BMAL1-mediated transcription (Chun et al., 2014). KS15 directly binds to CRY C-terminal domains, enhancing E-box-mediated transcription in a CRY-dependent manner, and attenuates the circadian oscillations of *Bmal1* and *Per2* promoter activity (Chun et al., 2014; Jang et al., 2018). Thus, while KL001 and its derivatives strengthen CRY-mediated feedback, KS15 increases basal promoter activity by inhibiting the repressive actions of CRYs on CLOCK/BMAL1-mediated transcription. CRYs are composed of highly conserved N-terminal photolyase homology regions and variable C-terminal extension domains (Chaves et al., 2006). The putative coiled-coil (CC) domain is located in the C-terminal tail and is highly conserved between CRY1 and CRY2. Previous studies have suggested that CRY C-terminal tails, including the CC domain, are important for nuclear localization and interactions with other core clock proteins (Chaves et al., 2006; van der Schalie et al., 2007). We found that KS15 binding of CRY C-terminal domains significantly inhibits CRY-BMAL1 interactions, but barely affects CRY-PER associations. Thus, KS15 can be used as a distinct scaffold to develop additional derivatives with improved pharmacokinetics, although further SAR studies are required to determine its mechanism of action (Jang et al., 2018).

### Circadian Nuclear Receptors as Small Molecule Probe Targets

The circadian nuclear receptors, REV-ERBs and RORs, mediate many physiological processes, including circadian rhythms, development, metabolism, immunity, and even various brain functions. Members of the nuclear receptor superfamily are ligand-activated transcription factors that act as intracellular receptors for cell-permeable ligands. Thus, nuclear receptors are considered as one of the primary molecular classes suitable for drug targets. Interestingly, REV-ERBs contain atypical ligand-binding domains (LBDs) and lack C-terminal transactivation domains, which are used for interactions with transcriptional co-activators. These features provide a structural basis for constitutively repressive action of REV-ERBs upon binding their target genes transcription. Recent studies have identified endogenous ligands for these circadian nuclear receptors, thereby stimulating the development of synthetic ligands with therapeutic applications to circadian rhythm-related diseases (Kojetin and Burris, 2014).

Although REV-ERBs were initially identified as orphan nuclear receptors, subsequent studies revealed that heme binds to the LBD of REV-ERBs (Raghuram et al., 2007; Yin et al., 2007). The discovery of endogenous REV-ERBs ligands led to the identification of chemical scaffolds that can act as synthetic ligands. The first identified synthetic REV-ERB ligand was GSK4112 (Meng et al., 2008). Specifically, GSK4112 is a REB-ERB agonist that enhances recruitment of NCoR and HDAC3 to their target promoters and then represses target gene transcription (Grant et al., 2010). While GSK4112 did not exhibit favorable pharmacokinetics, it paved the way for the subsequent development of synthetic REV-ERBs ligands. To improve potency, efficacy, and pharmacokinetics, Burris and colleagues developed additional REV-ERB agonists, such as SR9009 and SR9011, that were more suitable for *in vivo* applications. Both compounds demonstrated therapeutic efficacy of small molecule REV-ERB modulators in the treatment of circadian-related metabolic diseases and sleep disorders (Solt et al., 2012). Although there are several REV-ERBs agonists, SR8278 is the only antagonist that has been identified thus far. SR8278 inhibits the transcriptional repression activity of both REV-ERBs, thereby enhancing RRE-mediated transcription (Kojetin et al., 2011). So far, SR8278 applications *in vivo* have been limited; however, it provides a convenient tool to temporally inhibit REV-ERB activity in target cells or tissues.

Cholesterol and some of its metabolites were initially shown to act as natural ROR ligands. Recent studies revealed that several oxysterols are high-affinity endogenous ROR modulators. Oxysterol ligands bind directly to the RORα/γ LBD and act as inverse agonists by modulating the interaction of co-regulators. As indicated by their names, RORs are evolutionarily related to retinoic acid receptors. Interestingly, all-*trans* retinoic acids recognize the LBD of RORβ, but not RORα/γ, suggesting subtype specificity. Alternatively, the liver X receptor agonist, T0901317, was the first synthetic ligand and inverse agonist identified for RORα/γ (Kumar et al., 2010). Subsequently, a series of RORα/γ agonists or inverse agonists were developed as reviewed in more detail elsewhere (Kojetin and Burris, 2014). In a more recent study, Chen et al. identified that nobiletin, a natural polymethoxylated flavone, enhances circadian molecular rhythm amplitudes by acting on RORs (He et al., 2016).

## Implications in Circadian Rhythm-Related Neuropsychiatric Diseases

Considering the impact of the circadian system on a wide range of biological processes, small-molecule circadian modifiers may be used to optimize internal timing for pharmacological treatment and/or to rescue the desynchrony underlying circadian-related diseases. For example, SR9009/9011 and nobiletin have beneficial effects on high-fat diet-induced metabolic disturbances that affect a wide range of molecular, metabolic, and behavioral rhythms (Kohsaka et al., 2007; Solt et al., 2012; He et al., 2016). Dysregulation of circadian rhythmicity is also associated with various neuropsychiatric disorders, including sleep disorders, affective disorders, substance use disorders, schizophrenia, and neurodegeneration (Jagannath et al., 2013). However, determining whether the changes in brain function associated with these disorders manifest because of circadian dysregulation or additional malfunctions is controversial. Compelling evidence suggests that the effects of circadian disruption on brain function are attributable to both the SCN and local oscillators in discrete brain regions. Here, we will discuss how the circadian clock is involved in neuropsychiatric disorders and the potential implications of clock modulators for those diseases.

### Sleep Disorders

Given that the circadian system constitutes one of the two major mechanistic facets of sleep, small molecule clock modulators may be applicable for circadian rhythm-related sleep disorders. Indeed, abnormal sleep phenotypes have been reported in mutant mice with defective alleles of core clock genes as well as genes mediating post-translational modification of clock proteins (Sehgal and Mignot, 2011). For example, familial advanced sleep phase syndrome can be caused by either phosphorylation-defective mutations in human *PER2* or by mutant alleles for protein kinases such as CK1δ (Toh et al., 2001; Xu et al., 2005). A variation in human *PER3* is also associated with differential sleep homeostasis, particularly after sleep deprivation (Viola et al., 2007). Furthermore, REV-ERBs appear to have a certain role in both homeostatic and circadian regulation of sleep. In mutant mice with a defective allele of *Rev-erbα* gene, sleep/wake distributions are advanced in comparison with the environmental light-dark cycle. Moreover, both electroencephalogram delta power and sleep consolidation were also significantly reduced after sleep onset, suggesting a slower increase of homeostatic sleep need during wakefulness in the mutant mice (Mang et al., 2016). Interestingly, daytime administration of REV-ERB agonists induced wakefulness and suppressed both slow-wave and rapid eye movement sleep (Banerjee et al., 2014). Thus, pharmacological manipulation of the circadian clock may be used to treat circadian rhythm-related sleep disorders, such as sleep fragmentation, abnormal sleep phase syndromes, and non-24-h sleep-wake rhythm disorders.

### Mood-Related Psychiatric Disorders

Mood spectrum disorders, including major depression, bipolar disorder, seasonal affective disorder, and various addiction-related diseases, are the most attractive targets for clock modulators. Patients with mood disorders commonly suffer from disrupted sleep/wake cycles and dysregulated diurnal mood variations. Furthermore, several genetics studies have reported significant associations of clock genes with the onset and symptoms of affective disorders (Wulff et al., 2010; McCarthy and Welsh, 2012). Similarly, mutant mice with defective clock genes exhibit behavioral phenotypes linked with abnormal despair, anxiety, and reward responses (McClung et al., 2005; Hampp et al., 2008; Chung et al., 2014; Schnell et al., 2015). The key mediators of circadian mood regulation are central monoamine systems, making them ideal therapeutic targets for affective disorders. These monoamine systems are related to circadian disruption, as mutant mice bearing defective *ClockΔ19* demonstrate mania-like behaviors characterized by hyperactivity, as well as decreased depression- and anxiety-related behaviors. They also demonstrate increased cocaine sensitization with enhanced dopamine (DA) transmission (McClung et al., 2005). More recently, our data demonstrating mania-like phenotypes in *Rev-erbα*-deficient mice revealed that REV-ERBα connects the molecular clock with the midbrain DA system (Chung et al., 2014). CLOCK and REV-ERBα expression in DA neurons evokes daily variations in DA biosynthesis and transmission in mesocorticolimbic DA circuits, particularly through transcriptional control of tyrosine hydroxylase, a rate-limiting enzyme for catecholamine biosynthesis. Monoamine oxidase-mediated DA clearance in post-synaptic sites is also reported to be under the circadian control of NPAS2 and BMAL1 (Hampp et al., 2008). Taken together, these findings indicate that the circadian clock tightly controls DA biosynthesis, transmission, and turnover.

Interestingly, acute administration of the REV-ERB antagonist, SR8278, to the ventral midbrain produces mania-like behaviors with increased DA production and release (Chung et al., 2014). While both REV-ERB agonists and antagonists reduce anxiety-like behaviors in mice, REV-ERB agonists do not significantly affect despair-based behaviors (Banerjee et al., 2014; Chung et al., 2014). This discrepancy may arise from the presence of two REV-ERB isoforms that could interact with synthetic ligands. Although SR8278 promotes mania in wild-type mice, it acts as an anti-depressant in a mouse genetic model of depression (Guo et al., 2018). Nobiletin also has anti-depressant-like effects that are comparable with those of fluoxetine. These effects are also prevented by inhibitors for monoamine transmission (Yi et al., 2011). These findings strongly suggest that circadian clock modulators have therapeutic potential for mood-related psychiatric disorders, but also warn of potential risks in their clinical applications to circadian rhythm-related sleep and metabolic diseases.

### Neurodegenerative Diseases

Circadian disruptions are common among patients with neurodegenerative diseases, including Alzheimer’s disease (AD), Parkinson’s disease (PD), and Huntington’s disease, despite the range in pathogenesis and associated symptoms of these diseases (Hood and Amir, 2017). Circadian disturbances manifesting as alterations in sleep-wake cycles, hormone secretion, and diurnal mood regulation precede the cognitive and motor symptoms characteristic of these diseases. Indeed, various forms of AD models have exhibited phenotypes linked with circadian and/or sleep abnormalities (Wisor et al., 2005; Gorman and Yellon, 2010; Sterniczuk et al., 2010; Koss et al., 2016), and neurodegenerative lesions in the SCN have been proposed as a possible underlying mechanism (Sterniczuk et al., 2010; Zhou et al., 2016). Conversely, amyloid-beta (Aβ) pathologies are affected by the sleep-wake cycle in both mice and humans (Kang et al., 2009; Ooms et al., 2014). The sleep-wake cycle controls a diurnal rhythm found in Aβ levels in brain interstitial fluid (ISF) and sleep deprivation exacerbated Aβ plaque burden in an AD mouse model (Kang et al., 2009). These findings collectively suggest mutual interactions between circadian disturbances and neurodegenerative pathologies.

Considerable evidence suggests that circadian disturbances may play more direct roles in the progression of neurodegenerative diseases, particularly in sporadic disease forms (Musiek and Holtzman, 2016; Videnovic and Willis, 2016). Specifically, genetic variations in clock gene loci are associated with neurodegenerative diseases (Gu et al., 2015). Furthermore, the absence of functional BMAL1 is associated with various phenotypes of premature aging, increased oxidative stress, induced age-dependent gliosis, and neurodegeneration in the presence of neurotoxic assaults (Kondratov et al., 2006; Musiek et al., 2013). Recently, Musiek et al. (2013) demonstrated that loss of central circadian rhythms accelerates amyloid plaque accumulation along with disruption of daily Aβ oscillations in hippocampal ISF, whereas loss of local BMAL1 in extra-SCN brain regions promotes fibrillar plaque deposition and increased APOE expression, suggesting both central and local brain clock influence AD pathogenesis (Kress et al., 2018). It was also demonstrated that the expression of several AD risk genes, including *Bace1* and *Bace2*, are under the control of cellular clockworks (Ma et al., 2016). In addition to AD, genetic abrogation of REV-ERBα and chronic circadian disruption were shown to exacerbate neurotoxin-induced PD-like phenotypes and neuroinflammation-mediated DA neuron loss (Lauretti et al., 2017; Kim et al., 2018). Thus, chronic circadian disruption by either environmental or genetic causes is likely a risk factor for sporadic forms of neurodegenerative diseases, and neuroinflammatory dysregulation could be a link between circadian dysfunction and neurodegeneration (Musiek et al., 2013; Lauretti et al., 2017).

These findings also suggest that circadian rhythm-based therapeutics may delay the progression and severity of neurodegenerative diseases. Such chronobiological interventions for neuropsychiatric disorders, such as affective disorders and neurodegeneration, include bright-light therapy and timed melatonin administration (Forbes et al., 2014). Previous studies have suggested that timed light exposure and/or melatonin administration partially improve sleep- and circadian rhythm-related symptoms of AD and PD (Ancoli-Israel et al., 2003; Riemersma-van der Lek et al., 2008; Videnovic et al., 2017). However, whether bright light has long-lasting beneficial effects on cognitive or motor-skill impairments in AD or PD patients remains unclear. Alternatively, these impairments may be treated with small-molecule modulators of clock proteins. Indeed, nobiletin attenuated memory impairments and amyloid pathology in transgenic mouse models of AD (Onozuka et al., 2008; Nakajima et al., 2015) and ameliorated motor and cognitive deficits in MPTP-induced PD mice (Yabuki et al., 2014). Considering that clock proteins have been implicated in cellular antioxidant responses (Lee et al., 2013; Woldt et al., 2013), amelioration of oxidative damage may be an additional potential mechanism by which clock modulators delay neurodegeneration.

### Possible Mode of Actions: Brain Region-Specific and Systemic Mechanisms

Although still in preclinical development, small-molecule modifiers of clock components may exert beneficial effects at multiple levels (Sulli et al., 2018a). The simplest modes of action could involve cellular clock-dependent modulation of transcriptional networks or signaling pathways that are responsible for pathological states due to malfunction. Considering that the cellular clock coordinates diverse cellular pathways, pharmacological manipulation of clock components may have pleiotropic benefits, comparable to combination-therapy approaches. For example, a REV-ERBs antagonist enhanced both DA biosynthesis and activity-dependent neurotransmitter release, which may both contribute to its anti-depressant-like effects (Chung et al., 2014; Guo et al., 2018). In this context, it should be also noted that some molecular targets of established drugs have been identified to follow oscillatory expression or to be directly controlled by the cellular clock. Hogenesch et al. (1998) identified sets of oscillatory genes across multiple tissues in mice. Additionally, they found that more than 20% of the 100 best-selling drugs with short half-lives involved circadian gene targets; these drugs included widely used drugs for neuropsychiatric disorders such as insomnia, depressive disorders, and attention-deficit hyperactivity disorders (Zhang et al., 2014). These findings imply therapeutic potential of clock modulators that can be also considered for combinational therapy with existing treatments to improve their efficacy.

Systemic restoration or stabilization of the circadian system may also mediate therapeutic effects of clock modulators, plausibly by strengthening the autonomous oscillations of the SCN pacemaker and/or by helping the synchronization between brain clocks. As noted earlier, behavioral interventions to restore circadian rhythm and sleep have been reported to ameliorate some symptoms of affective disorders and neurodegeneration (Forbes et al., 2014; Sulli et al., 2018a). Furthermore, co-morbidities among circadian rhythm-related diseases are frequently found. For example, there is a bi-directional association between metabolic syndromes and mental health disorders including bipolar disorder, major depression, anxiety, attention-deficit hyperactivity disorder, schizophrenia, and autism spectrum disorders (Nousen et al., 2013). Because circadian rhythms coordinate the multiple brain systems responsible for affective, cognitive, and metabolic functions, dysregulation of circadian clocks has been proposed to play a central role in cardio-metabolic co-morbidity in psychiatric disorders (Barandas et al., 2015). It can be, therefore suggested that systemic actions of clock-targeting pharmaceuticals may provide additional distinct preventive or therapeutic strategies for co-morbid disorders.

## Concluding Remarks

Circadian clocks govern a wide spectrum of biochemical, physiological, and behavioral processes. Disruption or misalignment of the intrinsic rhythms are considered as a risk for the pathogenesis of various chronic diseases. Therefore, improving our understanding of the impact of the circadian system on brain functions may lead to the development of novel treatment schemes, increase efficacious therapeutic delivery, and improve preventative strategies for circadian rhythm-related brain disorders. In this context, development of chemical clock modulators may primarily contribute to revealing the functional relevance of the molecular clock across discrete brain regions because these small molecules can be used to dynamically control location-specific cellular clocks in the brain. More importantly, these molecules could provide lead structures for novel therapeutics for prevention and treatment of neuropsychiatric disorders.

## Author Contributions

All authors listed have made a substantial, direct and intellectual contribution to the work, and approved it for publication.

## Conflict of Interest Statement

The authors declare that the research was conducted in the absence of any commercial or financial relationships that could be construed as a potential conflict of interest.

## References

[B1] Ancoli-Israel S. Gehrman P. Martin J. L. Shochat T. Marler M. Corey-Bloom J. (2003). Increased light exposure consolidates sleep and strengthens circadian rhythms in severe Alzheimer’s disease patients. *Behav. Sleep Med.* 1 22–36. 10.1207/S15402010BSM0101-4 15600135

[B2] Asher G. Schibler U. (2006). A clock-less clock. *Trends Cell Biol.* 16 547–549. 10.1016/j.tcb.2006.09.005 16996737

[B3] Banerjee S. Wang Y. Solt L. A. Griffett K. Kazantzis M. Amador A. (2014). Pharmacological targeting of the mammalian clock regulates sleep architecture and emotional behaviour. *Nat. Commun.* 5:5759. 10.1038/ncomms6759 25536025 PMC4495958

[B4] Barandas R. Landgraf D. McCarthy M. J. Welsh D. K. (2015). Circadian clocks as modulators of metabolic comorbidity in psychiatric disorders. *Curr. Psychiatry Rep.* 17:98. 10.1007/s11920-015-0637-2 26483181

[B5] Bechtold D. A. Gibbs J. E. Loudon A. S. (2010). Circadian dysfunction in disease. *Trends Pharmacol. Sci.* 31 191–198. 10.1016/j.tips.2010.01.002 20171747

[B6] Billon C. Sitaula S. Burris T. P. (2016). Inhibition of RORα/γ suppresses atherosclerosis via inhibition of both cholesterol absorption and inflammation. *Mol. Metab.* 5 997–1005. 10.1016/j.molmet.2016.07.001 27689012 PMC5034492

[B7] Busino L. Bassermann F. Maiolica A. Lee C. Nolan P. M. Godinho S. I. (2007). SCFFbxl3 controls the oscillation of the circadian clock by directing the degradation of cryptochrome proteins. *Science* 316 900–904. 10.1126/science.1141194 17463251

[B8] Chaves I. Yagita K. Barnhoorn S. Okamura H. van der Horst G. T. Tamanini F. (2006). Functional evolution of the photolyase/cryptochrome protein family importance of the C terminus of mammalian CRY1 for circadian core oscillator performance. *Mol. Cell. Biol.* 26 1743–1753. 10.1128/MCB.26.5.1743-1753.2006 16478995 PMC1430250

[B9] Chen Z. Yoo S. H. Takahashi J. S. (2018). Development and therapeutic potential of small-molecule modulators of circadian systems. *Annu. Rev. Pharmacol. Toxicol.* 58 231–252. 10.1146/annurev-pharmtox-010617-052645 28968186 PMC6076890

[B10] Cho H. Zhao X. Hatori M. Yu R. T. Barish G. D. Lam M. T. (2012). Regulation of circadian behaviour and metabolism by REV-ERB-α and REV-ERB-β. *Nature* 485 123–127. 10.1038/nature11030 22460952 PMC3367514

[B11] Chun S. K. Chung S. Kim H. D. Lee J. H. Jang J. Kim J. (2015). A synthetic cryptochrome inhibitor induces anti-proliferative effects and increases chemosensitivity in human breast cancer cells. *Biochem. Biophys. Res. Commun.* 467 441–446. 10.1016/j.bbrc.2015.09.103 26407844

[B12] Chun S. K. Jang J. Chung S. Yun H. Kim N. J. Jung J. W. (2014). Identification and validation of cryptochrome inhibitors that modulate the molecular circadian clock. *ACS Chem. Biol.* 9 703–710. 10.1021/cb400752k 24387302

[B13] Chung S. Lee E. J. Yun S. Choe H. K. Park S. B. Son H. J. (2014). Impact of circadian nuclear receptor REV-ERBα on midbrain dopamine production and mood regulation. *Cell* 157 858–868. 10.1016/j.cell.2014.03.039 24813609

[B14] Dai J. Choo M. K. Park J. M. Fisher D. E. (2017). Topical ROR inverse agonists suppress inflammation in mouse models of atopic dermatitis and acute irritant dermatitis. *J. Invest. Dermatol.* 137 2523–2531. 10.1016/j.jid.2017.07.819 28774591 PMC5990371

[B15] Dibner C. Schibler U. Albrecht U. (2010). The mammalian circadian timing system: organization and coordination of central and peripheral clocks. *Annu. Rev. Physiol.* 72 517–549. 10.1146/annurev-physiol-021909-135821 20148687

[B16] Eide E. J. Woolf M. F. Kang H. Woolf P. Hurst W. Camacho F. (2005). Control of mammalian circadian rhythm by CKIepsilon-regulated proteasome-mediated PER2 degradation. *Mol. Cell. Biol.* 25 2795–2807. 10.1128/MCB.25.7.2795-2807.2005 15767683 PMC1061645

[B17] Forbes D. Blake C. M. Thiessen E. J. Peacock S. Hawranik P. (2014). Light therapy for improving cognition, activities of daily living, sleep, challenging behaviour and psychiatric disturbances in dementia. *Cochrane Database Syst. Rev.* 26:CD003946. 10.1002/14651858.CD003946.pub4 24574061 PMC10837684

[B18] Gekakis N. Staknis D. Nguyen H. B. Davis F. C. Wilsbacher L. D. King D. P. (1998). Role of the CLOCK protein in the mammalian circadian mechanism. *Science* 280 564–569. 10.1126/science.280.5369.1564 9616112

[B19] Godinho S. I. Maywood E. S. Shaw L. Tucci V. Barnard A. R. Busino L. (2007). The after-hours mutant reveals a role for Fbxl3 in determining mammalian circadian period. *Science* 316 897–900. 10.1126/science.1141138 17463252

[B20] Gorman M. R. Yellon S. (2010). Lifespan daily locomotor activity rhythms in a mouse model of amyloid-induced neuropathology. *Chronobiol. Int.* 27 1159–1177. 10.3109/07420528.2010.485711 20653448

[B21] Grant D. Yin L. Collins J. L. Parks D. J. Orband-Miller L. A. Wisely G. B. (2010). GSK4112, a small molecule chemical probe for the cell biology of the nuclear heme receptor Rev-erbα. *ACS Chem. Biol.* 5 925–932. 10.1021/cb100141y 20677822

[B22] Gu Z. Wang B. Zhang Y. B. Ding H. Zhang Y. Yu J. (2015). Association of ARNTL and PER1 genes with Parkinson’s disease: a case-control study of han chinese. *Sci. Rep.* 5:15891. 10.1038/srep15891 26507264 PMC4623766

[B23] Guo D. Zhang S. Sun H. Xu X. Hao Z. Mu C. (2018). Tyrosine hydroxylase down-regulation after loss of Abelson helper integration site 1 (AHI1) promotes depression via the circadian clock pathway in mice. *J. Biol. Chem.* 293 5090–5101. 10.1074/jbc.RA117.000618 29449373 PMC5892572

[B24] Hampp G. Ripperger J. A. Houben T. Schmutz I. Blex C. Perreau-Lenz S. (2008). Regulation of monoamine oxidase A by circadian-clock components implies clock influence on mood. *Curr. Biol.* 18 678–683. 10.1016/j.cub.2008.04.012 18439826

[B25] He B. Chen Z. (2016). Molecular targets for small-molecule modulators of circadian clocks. *Curr. Drug Metab.* 17 503–512. 10.2174/1389200217666160111124439 26750111 PMC4825319

[B26] He B. Nohara K. Park N. Park Y. S. Guillory B. Zhao Z. (2016). The small molecule nobiletin targets the molecular oscillator to enhance circadian rhythms and protect against metabolic syndrome. *Cell Metab.* 23 610–621. 10.1016/j.cmet.2016.03.007 27076076 PMC4832569

[B27] Hirano A. Yumimoto K. Tsunematsu R. Matsumoto M. Oyama M. Kozuka-Hata H. (2013). FBXL21 regulates oscillation of the circadian clock through ubiquitination and stabilization of cryptochromes. *Cell* 152 1106–1118. 10.1016/j.cell.2013.01.054 23452856

[B28] Hirota T. Lee J. W. St. John P. C. Sawa M. Iwaisako K. Noguchi T. (2012). Identification of small molecule activators of cryptochrome. *Science* 337 1094–1097. 10.1126/science.1223710 22798407 PMC3589997

[B29] Hirota T. Lewis W. G. Liu A. C. Lee J. W. Schultz P. G. Kay S. A. (2008). A chemical biology approach reveals period shortening of the mammalian circadian clock by specific inhibition of GSK-3beta. *Proc. Natl. Acad. Sci. U.S.A.* 105 20746–20751. 10.1073/pnas.0811410106 19104043 PMC2606900

[B30] Hogenesch J. B. Gu Y. Z. Jain S. Bradfield C. A. (1998). The basic-helix-loop-helix-PAS orphan MOP3 forms transcriptionally active complexes with circadian and hypoxia factors. *Proc. Natl Acad. Sci. U.S.A.* 95 5474–5479. 10.1073/pnas.95.10.5474 9576906 PMC20401

[B31] Honma S. Kawamoto T. Takagi Y. Fujimoto K. Sato F. Noshiro M. (2002). Dec1 and Dec2 are regulators of the mammalian molecular clock. *Nature* 419 841–844. 10.1038/nature01123 12397359

[B32] Hood S. Amir S. (2017). Neurodegeneration and the circadian clock. *Front. Aging Neurosci.* 9:170 10.3389/fnagi.2017.00170 PMC5447688 28611660

[B33] Jagannath A. Peirson S. N. Foster R. G. (2013). Sleep and circadian rhythm disruption in neuropsychiatric illness. *Curr. Opin. Neurobiol.* 23 888–894. 10.1016/j.conb.2013.03.008 23618559

[B34] Jang J. Chung S. Choi Y. Lim H. Y. Son Y. Chun S. K. (2018). The cryptochrome inhibitor KS15 enhances E-box-mediated transcription by disrupting the feedback action of a circadian transcription-repressor complex. *Life Sci.* 200 49–55. 10.1016/j.lfs.2018.03.022 29534992

[B35] Kang J. E. Lim M. M. Bateman R. J. Lee J. J. Smyth L. P. Cirrito J. R. (2009). Amyloid-beta dynamics are regulated by orexin and the sleep-wake cycle. *Science* 326 1005–1007. 10.1126/science.1180962 19779148 PMC2789838

[B36] Kim J. Jang S. Choi M. Chung S. Choe Y. Choe H. K. (2018). Abrogation of the circadian nuclear receptor REV-ERBα exacerbates 6-hydroxydopamine-induced dopaminergic neurodegeneration. *Mol. Cells* 41 742–752. 10.14348/molcells.2018.0201 30078232 PMC6125424

[B37] Kohsaka A. Laposky A. D. Ramsey K. M. Estrada C. Joshu C. Kobayashi Y. (2007). High-fat diet disrupts behavioral and molecular circadian rhythms in mice. *Cell Metab.* 6 414–421. 10.1016/j.cmet.2007.09.006 17983587

[B38] Kojetin D. Wang Y. Kamenecka T. M. Burris T. P. (2011). Identification of SR8278, a synthetic antagonist of the nuclear heme receptor REV-ERB. *ACS Chem. Biol.* 6 131–134. 10.1021/cb1002575 21043485 PMC3042041

[B39] Kojetin D. J. Burris T. P. (2014). REV-ERB and ROR nuclear receptors as drug targets. *Nat. Rev. Drug Discov.* 13 197–216. 10.1038/nrd4100 24577401 PMC4865262

[B40] Kondratov R. V. Kondratova A. A. Gorbacheva V. Y. Vykhovanets O. V. Antoch M. P. (2006). Early aging and age-related pathologies in mice deficient in BMAL1, the core componentof the circadian clock. *Genes Dev.* 20 1868–1873. 10.1101/gad.1432206 16847346 PMC1522083

[B41] Koss D. J. Robinson L. Drever B. D. Pluciǹska K. Stoppelkamp S. Veselcic P. (2016). Mutant Tau knock-in mice display frontotemporal dementia relevant behaviour and histopathology. *Neurobiol. Dis.* 91 105–123. 10.1016/j.nbd.2016.03.002 26949217

[B42] Kress G. J. Liao F. Dimitry J. Cedeno M. R. FitzGerald G. A. Holtzman D. M. (2018). Regulation of amyloid-β dynamics and pathology by the circadian clock. *J. Exp. Med.* 215 1059–1068. 10.1084/jem.20172347 29382695 PMC5881473

[B43] Kumar N. Solt L. A. Conkright J. J. Wang Y. Istrate M. A. Busby S. A. (2010). The benzenesulfoamide T0901317 [N-(2,2,2-trifluoroethyl)-N-[4-[2,2,2-trifluoro-1-hydroxy-1-(trifluoromethyl)ethyl]phenyl]-benzenesulfonamide] is a novel retinoic acid receptor-related orphan receptor-alpha/gamma inverse agonist. *Mol. Pharmacol.* 77 228–236. 10.1124/mol.109.060905 19887649 PMC2812071

[B44] Kume K. Zylka M. J. Sriram S. Shearman L. P. Weaver D. R. Jin X. (1999). mCRY1 and mCRY2 are essential components of the negative limb of the circadian clock feedback loop. *Cell* 98 193–205. 10.1016/S0092-8674(00)81014-4 10428031

[B45] Kurabayashi N. Hirota T. Sakai M. Sanada K. Fukada Y. (2010). DYRK1A and glycogen synthase kinase 3beta, a dual-kinase mechanism directing proteasomal degradation of CRY2 for circadian timekeeping. *Mol. Cell. Biol.* 30 1757–1768. 10.1128/MCB.01047-09 20123978 PMC2838083

[B46] Lamia K. A. Sachdeva U. M. DiTacchio L. Williams E. C. Alvarez J. G. Egan D. F. (2009). AMPK regulates the circadian clock by cryptochrome phosphorylation and degradation. *Science* 326 437–440. 10.1126/science.1172156 19833968 PMC2819106

[B47] Lauretti E. Di Meco A. Merali S. Praticò D. (2017). Circadian rhythm dysfunction: a novel environmental risk factor for Parkinson’s disease. *Mol. Psychiatry* 22 280–286. 10.1038/mp.2016.47 27046648

[B48] Lee J. Moulik M. Fang Z. Saha P. Zou F. Xu Y. (2013). Bmal1 and β-cell clock are required for adaptation to circadian disruption and their loss of function leads to oxidative stress-induced b-cell failure in mice. *Mol. Cell. Biol.* 33 2327–2338. 10.1128/MCB.014210-12 23547261 PMC3648066

[B49] Li J. Lu W. Q. Beesley S. Loudon A. S. Meng Q. J. (2012). Lithium impacts on the amplitude and period of the molecular circadian clockwork. *PLoS One* 7:e33292. 10.1371/journal.pone.0033292 22428012 PMC3299767

[B50] Ma Z. Jiang W. Zhang E. E. (2016). Orexin signaling regulates both the hippocampal clock and the circadian oscillation of Alzheimer’s disease-risk genes. *Sci. Rep.* 6:36035. 10.1038/srep36035 27796320 PMC5086843

[B51] Mang G. M. La Spada F. Emmenegger Y. Chappuis S. Ripperger J. A. Albrecht U. (2016). Altered sleep homeostasis in rev-erbα knockout mice. *Sleep* 39 589–601. 10.5665/sleep.5534 26564124 PMC4763348

[B52] McCarthy M. J. Welsh D. K. (2012). Cellular circadian clocks in mood disorders. *J. Biol. Rhythms* 27 339–352. 10.1177/0748730412456367 23010657

[B53] McClung C. A. Sidiropoulou K. Vitaterna M. Takahashi J. S. White F. J. Cooper D. C. (2005). Regulation of dopaminergic transmission and cocaine reward by the Clock gene. *Proc. Natl. Acad. Sci. U.S.A.* 102 9377–9381. 10.1073/pnas.0503584102 15967985 PMC1166621

[B54] Meng Q. J. McMaster A. Beesley S. Lu W. Q. Gibbs J. Parks D. (2008). Ligand modulation of REV-ERBalpha function resets the peripheral circadian clock in a phasic manner. *J. Cell. Sci.* 121 3629–3635. 10.1242/jcs.035048 18946026 PMC3069549

[B55] Mitsui S. Yamaguchi S. Matsuo T. Ishida Y. Okamura H. (2001). Antagonistic role of E4BP4 and PAR proteins in the circadian oscillatory mechanism. *Genes Dev.* 15 995–1006. 10.1101/gad.873501 11316793 PMC312673

[B56] Musiek E. S. Holtzman D. M. (2016). Mechanisms linking circadian clocks, sleep, and neurodegeneration. *Science* 354 1004–1008. 10.1126/science.aah4968 27885006 PMC5219881

[B57] Musiek E. S. Lim M. M. Yang G. Bauer A. Q. Qi L. Lee Y. (2013). Circadian clock proteins regulate neuronal redox homeostasis and neurodegeneration. *J. Clin. Invest.* 123 5389–5400. 10.1172/JCI70317 24270424 PMC3859381

[B58] Nakajima A. Aoyama Y. Shin E.-J. Nam Y. Kim H.-C. Nagai T. (2015). Nobiletin, a citrus flavonoid, improves cognitive impairment and reduces soluble ABeta levels in a triple transgenic mouse model of Alzheimer’s disease. *Behav. Brain Res.* 289 69–77. 10.1016/j.bbr.2015.04.028 25913833

[B59] Nangle S. Xing W. Zheng N. (2013). Crystal structure of mammalian cryptochrome in complex with a small molecule competitor of its ubiquitin ligase. *Cell Res.* 23 1417–1419. 10.1038/cr.2013.136 24080726 PMC3847572

[B60] Nousen E. K. Franco J. G. Sullivan E. L. (2013). Unraveling the mechanisms responsible for the comorbidity between metabolic syndrome and mental health disorders. *Neuroendocrinology* 98 254–266. 10.1159/000355632 24080959 PMC4121390

[B61] Onozuka H. Nakajima A. Matsuzaki K. Shin R.-W. Ogino K. Saigusa D. (2008). Nobiletin, a citrus flavonoid, improves memory impairment and ABeta pathology in a transgenic mouse model of Alzheimer’s disease. *J. Pharmacol. Exp. Ther.* 326 739–744. 10.1124/jpet.108.140293 18544674

[B62] Ooms S. Overeem S. Besse K. Rikkert M. O. Verbeek M. Claassen J. A. (2014). Effect of 1 night of total sleep deprivation on cerebrospinal fluid β-amyloid 42 in healthy middle-aged men: a randomized clinical trial. *JAMA Neurol.* 71 971–977. 10.1001/jamaneurol.2014.1173 24887018

[B63] Preitner N. Damiola F. Lopez-Molina L. Zakany J. Duboule D. Albrecht U. (2002). The orphan nuclear receptor REV-ERBalpha controls circadian transcription within the positive limb of the mammalian circadian oscillator. *Cell* 110 251–260. 10.1016/S0092-8674(02)00825-5 12150932

[B64] Raghuram S. Stayrook K. R. Huang P. Rogers P. M. Nosie A. K. McClure D. B. (2007). Identification of heme as the ligand for the orphan nuclear receptors REV-ERBalpha and REV-ERBbeta. *Nat. Struct. Mol. Biol.* 14 1207–1213. 10.1038/nsmb1344 18037887 PMC2743565

[B65] Ralph M. R. Foster R. G. Davis F. C. Menaker M. (1990). Transplanted suprachiasmatic nucleus determines circadian period. *Science* 247 975–978. 10.1126/science.2305266 2305266

[B66] Ralph M. R. Menaker M. (1988). A mutation of the circadian system in golden hamsters. *Science* 241 1225–1227. 10.1126/science.3413487 3413487

[B67] Reppert S. M. Weaver D. R. (2002). Coordination of circadian timing in mammals. *Nature* 418 935–941. 10.1038/nature00965 12198538

[B68] Riemersma-van der Lek R. F. Swaab D. F. Twisk J. Hol E. M. Hoogendijk W. J. Van Someren E. J. (2008). Effect of bright light and melatonin on cognitive and noncognitive function in elderly residents of group care facilities: a randomized controlled trial. *JAMA* 299 2642–2655. 10.1001/jama.299.22.2642 18544724

[B69] Sato T. K. Panda S. Miraglia L. J. Reyes T. M. Rudic R. D. McNamara P. (2004). A functional genomics strategy reveals Rora as a component of the mammalian circadian clock. *Neuron* 43 527–537. 10.1016/j.neuron.2004.07.018 15312651

[B70] Schnell A. Sandrelli F. Ranc V. Ripperger J. A. Brai E. Alberi L. (2015). Mice lacking circadian clock components display different mood-related behaviors and do not respond uniformly to chronic lithium treatment. *Chronobiol. Int.* 32 1075–1089. 10.3109/07420528.2015.1062024 26317159

[B71] Schroeder A. M. Colwell C. S. (2013). How to fix a broken clock. *Trends Pharmacol. Sci.* 34 605–619. 10.1016/j.tips.2013.09.002 24120229 PMC3856231

[B72] Sehgal A. Mignot E. (2011). Genetics of sleep and sleep disorders. *Cell* 146 194–207. 10.1016/j.cell.2011.07.004 21784243 PMC3153991

[B73] Shearman L. P. Sriram S. Weaver D. R. Maywood E. S. Chaves I. Zheng B. (2000). Interacting molecular loops in the mammalian circadian clock. *Science* 288 1013–1019. 10.1126/science.288.5468.1013 10807566

[B74] Shirogane T. Jin J. Ang X. L. Harper J. W. (2005). SCFbeta-TRCP controls clock-dependent transcription via casein kinase 1-dependent degradation of the mammalian period-1 (Per1) protein. *J. Biol. Chem.* 280 26863–26872. 10.1074/jbc.M502862200 15917222

[B75] Siepka S. M. Yoo S. H. Park J. Song W. Kumar V. Hu Y. (2007). Circadian mutant overtime reveals F-box protein FBXL3 regulation of cryptochrome and period gene expression. *Cell* 129 1011–1023. 10.1016/j.cell.2007.04.030 17462724 PMC3762874

[B76] Solt L. A. Kumar N. Nuhant P. Wang Y. Lauer J. L. Liu J. (2011). Suppression of TH17 differentiation and autoimmunity by a synthetic ROR ligand. *Nature* 472 491–494. 10.1038/nature10075 21499262 PMC3148894

[B77] Solt L. A. Wang Y. Banerjee S. Hughes T. Kojetin D. J. Lundasen T. (2012). Regulation of circadian behaviour and metabolism by synthetic REV-ERB agonists. *Nature* 485 62–68. 10.1038/nature11030 22460951 PMC3343186

[B78] Son G. H. Chung S. Kim K. (2011). The adrenal peripheral clock: glucocorticoid and the circadian timing system. *Front. Neuroendocrinol.* 32:451–465. 10.1016/j.yfrne.2011.07.003 21802440

[B79] Sterniczuk R. Dyck R. H. Laferla F. M. Antle M. C. (2010). Characterization of the 3xTg-AD mouse model of Alzheimer’s disease: part 1. circadian changes. *Brain Res.* 1348 139–148. 10.1016/j.brainres.2010.05.013 20471965

[B80] Sulli G. Manoogian E. N. C. Taub P. R. Panda S. (2018a). Training the circadian clock, clocking the drugs, and drugging the clock to prevent, manage, and treat chronic diseases. *Trends Pharmacol. Sci.* 39 812–827. 10.1016/j.tips.2018.07.003 30060890 PMC7249726

[B81] Sulli G. Rommel A. Wang X. Kolar M. J. Puca F. Saghatelian A. (2018b). Pharmacological activation of REV-ERBs is lethal in cancer and oncogene-induced senescence. *Nature* 553 351–355. 10.1038/nature25170 29320480 PMC5924733

[B82] Takahashi J. S. Hong H. K. Ko C. H. McDearmon E. L. (2008). The genetics of mammalian circadian order and disorder: implications for physiology and disease. *Nat. Rev. Genet.* 9 764–775. 10.1038/nrg2430 18802415 PMC3758473

[B83] Toh K. L. Jones C. R. He Y. Eide E. J. Hinz W. A. Virshup D. M. (2001). An hPer2 phosphorylation site mutation in familial advanced sleep phase syndrome. *Science* 291 1040–1043. 10.1126/science.1057499 11232563

[B84] Ueda H. R. Chen W. Adachi A. Wakamatsu H. Hayashi S. Takasugi T. (2002). A transcription factor response element for gene expression during circadian night. *Nature* 418 534–539. 10.1038/nature00906 12152080

[B85] Um J. H. Yang S. Yamazaki S. Kang H. Viollet B. Foretz M. (2007). Activation of 5’-AMP-activated kinase with diabetes drug metformin induces casein kinase Iepsilon (CKIepsilon)-dependent degradation of clock protein mPer2. *J. Biol. Chem.* 282 20794–20798. 10.1074/jbc.C700070200 17525164

[B86] van der Schalie E. A. Conte F. E. Marz K. E. Green C. B. (2007). Structure/function analysis of xenopus cryptochromes 1 and 2 reveals differential nuclear localization mechanisms and functional domains important for interaction with and repression of CLOCKBMAL1. *Mol. Cell. Biol.* 27 2120–2129. 10.1128/MCB.01638-06 17210647 PMC1820510

[B87] Videnovic A. Klerman E. B. Wang W. Marconi A. Kuhta T. Zee P. C. (2017). Timed light therapy for sleep and daytime sleepiness associated with Parkinson disease: a randomized clinical trial. *JAMA Neurol.* 74 411–418. 10.1001/jamaneurol.2016.5192 28241159 PMC5470356

[B88] Videnovic A. Willis G. L. (2016). Circadian system - a novel diagnostic and therapeutic target in Parkinson’s disease? *Mov. Disord.* 31 260–269. 10.1002/mds.26509 26826022 PMC4783245

[B89] Viola A. U. Archer S. N. James L. M. Groeger J. A. Lo J. C. Skene D. J. (2007). PER3 polymorphism predicts sleep structure and waking performance. *Curr. Biol.* 17 613–618. 10.1016/j.cub.2007.01.073 17346965

[B90] Wang Y. Billon C. Walker J. K. Burris T. P. (2016). Therapeutic effect of a synthetic RORα/γ agonist in an animal model of autism. *ACS Chem. Neurosci.* 7 143–148. 10.1021/acschemneuro.5b00159 26625251 PMC4759619

[B91] Wang Y. Kumar N. Nuhant P. Cameron M. D. Istrate M. A. Roush W. R. (2010). Identification of SR1078, a synthetic agonist for the orphan nuclear receptors RORα and RORγ. *ACS Chem. Biol.* 5 1029–1034. 10.1021/cb100223d 20735016 PMC3003750

[B92] Wisor J. P. Edgar D. M. Yesavage J. Ryan H. S. McCormick C. M. Lapustea N. (2005). Sleep and circadian abnormalities in a transgenic mouse model of Alzheimer’s disease: a role for cholinergic transmission. *Neuroscience* 131 375–385. 10.1016/j.neuroscience.2004.11.018 15708480

[B93] Woldt E. Sebti Y. Solt L. A. Duhem C. Lancel S. Eeckhoute J. (2013). Rev-erb-α modulates skeletal muscle oxidative capacity by regulating mitochondrial biogenesis and autophagy. *Nat. Med.* 19 1039–1046. 10.1038/nm.3213 23852339 PMC3737409

[B94] Wulff K. Gatti S. Wettstein J. G. Foster R. G. (2010). Sleep and circadian rhythm disruption in psychiatric and neurodegenerative disease. *Nat. Rev. Neurosci.* 11 589–599. 10.1038/nrn2868 20631712

[B95] Xing W. Busino L. Hinds T. R. Marionni S. T. Saifee N. H. Bush M. F. (2013). SCF(FBXL3) ubiquitin ligase targets cryptochromes at their cofactor pocket. *Nature* 496 64–68. 10.1038/nature11964 23503662 PMC3618506

[B96] Xu Y. Padiath Q. S. Shapiro R. E. Jones C. R. Wu S. C. Saigoh N. (2005). Functional consequences of a CKIdelta mutation causing familial advanced sleep phase syndrome. *Nature* 434 640–644. 10.1038/nature03453 15800623

[B97] Yabuki Y. Ohizumi Y. Yokosuka A. Mimaki Y. Fukunaga K. (2014). Nobiletin treatment improves motor and cognitive deficits seen in MPTP-induced Parkinson model mice. *Neuroscience* 259 126–141. 10.1016/j.neuroscience.2013.11.051 24316474

[B98] Yi L. T. Xu H. L. Feng J. Zhan X. Zhou L. P. Cui C. C. (2011). Involvement of monoaminergic systems in the antidepressant-like effect of nobiletin. *Physiol. Behav.* 102 1–6. 10.1016/j.physbeh.2010.10.008 20951716

[B99] Yin L. Wu N. Curtin J. C. Qatanani M. Szwergold N. R. Reid R. A. (2007). Rev-erbalpha, a heme sensor that coordinates metabolic and circadian pathways. *Science* 318 1786–1789. 10.1126/science.1150179 18006707

[B100] Yoo S. H. Mohawk J. A. Siepka S. M. Shan Y. Huh S. K. Hong H. K. (2013). Competing E3 ubiquitin ligases govern circadian periodicity by degradation of CRY in nucleus and cytoplasm. *Cell* 152 1091–1105. 10.1016/j.cell.2013.01.055 23452855 PMC3694781

[B101] Zhang R. Lahens N. F. Balance H. I. Hughes M. E. Hogenesch J. B. (2014). A circadian gene expression atlas in mammals: implications for biology and medicine. *Proc. Natl. Acad. Sci. U.S.A.* 111 16219–16224. 10.1073/pnas.1408886111 25349387 PMC4234565

[B102] Zhang Y. Fang B. Emmett M. J. Damle M. Sun Z. Feng D. (2015). Discrete functions of nuclear receptor Rev-erbα couple metabolism to the clock. *Science* 348 1488–1492. 10.1126/science.aab3021 26044300 PMC4613749

[B103] Zhao Y. Xu L. Ding S. Lin N. Ji Q. Gao L. (2017). Novel protective role of the circadian nuclear receptor retinoic acid-related orphan receptor-α in diabetic cardiomyopathy. *J. Pineal Res.* 62:e12378. 10.1111/jpi.12378 27862268

[B104] Zhou L. Gao Q. Nie M. Gu J. L. Hao W. Wang L. (2016). Degeneration and energy shortage in the suprachiasmatic nucleus underlies the circadian rhythm disturbance in ApoE-/- mice: implications for Alzheimer’s disease. *Sci. Rep.* 6:36335. 10.1038/srep36335 27824104 PMC5099891

